# Amino acid residues L261, W264, F265, L268, and V269 of apolipoprotein E4 critically regulate adipose tissue metabolism

**DOI:** 10.1016/j.jlr.2025.100958

**Published:** 2025-12-05

**Authors:** Evangelia Zvintzou, Panagiota C. Giannopoulou, Katerina Giannatou, Radu Ionita, Maria Alemi, Georgia Kakafoni, Victoria Mparnia, Madalina Dumitrescu, Ioana Madalina Fenyo, Anca Violeta Gafencu, Kyriakos E. Kypreos

**Affiliations:** 1 Pharmacology Laboratory Department of Medicine, University of Patras, Rio Achaias, Greece; 2 Institute of Cellular Biology and Pathology, “Nicolae Simionescu” of the Romanian Academy, Bucharest, Romania; 3 Department of Life Sciences, School of Sciences, European University Cyprus, Nicosia, Cyprus

**Keywords:** adipose tissue, apolipoprotein E, APOE4mut1, morbid obesity, mitochondrial metabolism

## Abstract

Apolipoprotein E (APOE) plays a tissue-specific role in diet-induced obesity: brain-expressed APOE promotes obesity, while hepatic APOE appears protective. Physiological plasma APOE levels facilitate clearance of atherogenic lipoproteins; however, supraphysiological levels induce hypertriglyceridemia and impair cholesterol clearance. APOE-induced hypertriglyceridemia has been linked to its carboxyl-terminal region (amino acids 260–270), particularly residues L261, W264, F265, L268, and V269. A bioengineered APOE4 variant, APOE4mut1, where these residues are substituted with alanine, promotes cholesterol clearance without inducing hypertriglyceridemia at any level of expression. This study examined APOE4mut1 effects on adipose tissue metabolism in vivo. Wild-type (C57BL/6) and APOE4_knock-in_ mice were fed a Western-type diet for varying periods and infected with adenoviruses expressing *APOE4* (AdAPOE4), *APOE4mut1* (AdAPOE4mut1), or only the green fluorescent protein (GFP) (AdGFP). AdAPOE4mut1 infection of C57BL/6 mice fed a Western-type diet for 8 or 24 weeks stimulated brown adipose tissue (BAT) metabolism by inducing non-shivering thermogenesis and oxidative phosphorylation. In contrast, AdAPOE4 suppressed thermogenesis in this tissue. In white adipose tissue (WAT), AdAPOE4mut1 was able to stimulate thermogenesis after 24 weeks of feeding. This stimulatory effect in WAT was dominant over wild-type APOE4, since APOE4mut1 similarly enhanced mitochondrial activity in WAT of APOE4_knock-in_ mice. These findings suggest that amino acid residues 260–270 of APOE4 critically regulate adipose tissue metabolism, in addition to their previously reported role in APOE-induced hypertriglyceridemia. Targeted mutagenesis within this region offers a potential therapeutic strategy for addressing hypertriglyceridemia and obesity in metabolic syndrome.

## Graphical Abstract

**Figure:**
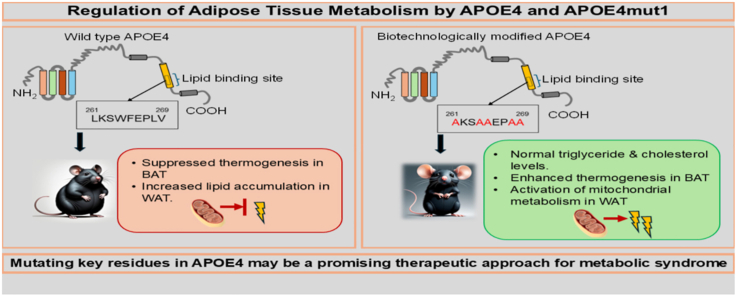

Morbid obesity and its metabolic perturbations are increasing at an alarming pace worldwide, posing a major social and economic challenge for patients, their immediate family and the health care systems around the globe. Unfortunately, nutritional and lifestyle interventions and current pharmacological and surgical treatments fall short of their mission due to numerous efficacy and safety issues (1, 2). Morbid obesity is the result of dysfunctional adipose tissue, which in turn results in hormonal imbalance and irregular adipokine secretion (3).

Adipose tissue consists of white adipose tissue (WAT) which is mainly responsible for lipid storage, and brown adipose tissue (BAT) which is mainly responsible for energy production (heat and ATP). Under certain circumstances, WAT may be activated metabolically and turn into BRITE (BRown Into whiTE) adipose tissue that is able to produce heat via non-shivering thermogenesis but localizes in WAT depots (4). The origin of newly formed brown adipocytes may be the result of transdifferentiation of white adipocytes into brown (5). Increased non-shivering thermogenesis is a result of elevated mitochondrial metabolic activity and mainly uncoupling protein 1 (Ucp1) function that mediates the metabolic conversion of free fatty acids to heat, thus contributing to the lean phenotype (6, 7). However, induction of WAT mitochondrial oxidative phosphorylation for ATP production, independent of Ucp1 increase, may also contribute to the lean phenotype (8). Metabolic activation of WAT into BRITE remains a promising strategy for the treatment of morbid obesity. Recent studies in mice strongly suggest a link between apolipoprotein E (APOE) and morbid obesity (9, 10, 11, 12). Similarly, we recently reported that APOE plays a tissue-specific role in diet-induced obesity: Brain-expressed APOE promotes obesity, while hepatically expressed APOE appears protective (13).

APOE is a glycoprotein synthesized in the liver brain, intestines, adipose tissue, spleen, and kidneys (14, 15). APOE plays a crucial role in lipid metabolism and is found in humans in three natural isoforms, APOE2, APOE3 and APOE4, consisting of 299 amino acids with a molecular weight of 34 kDa. These natural isoforms of APOE differ at positions 112 and 158, with APOE2 containing Cys/Cys, APOE3 containing Cys/Arg and APOE4 containing Arg/Arg (16). Structurally, murine APOE resembles the human APOE4 isoform. Functionally, however, it is closer to human APOE3 (17, 18). Crystallographic studies of APOE have revealed a sophisticated structural organization, with the amino-terminal end (residues 1–191) forming four amphipathic helices arranged in an antiparallel configuration, while the carboxy-terminal portion (residues 216–299) contains three α-helices (19). These two regions are connected by a flexible hinge region (residues 192–215), which may be critical for the protein's conformational flexibility and its functional interactions with other molecules (20).

In experimental models, it has been shown that the region spanning amino acids 1–185 of APOE mediates the binding of the protein to lipoproteins and the interaction with the low-density lipoprotein receptor (LDLR), facilitating clearance of Apolipoprotein B (APOB)-containing lipoproteins (21, 22). The fourth helix, particularly the amino acid region between residues 130–160, contains the LDLR binding site and is essential for APOE’s role in lipid transport and metabolism. The carboxy-terminal region spanning residues 260–270, specifically residues L261, W264, F265, L268, and V269 in helix 8, is responsible for inhibiting lipoprotein lipase (LpL) and promoting hepatic very low density lipoprotein (VLDL)-triglyceride secretion (23, 24). This composite effect on triglycerides has significant consequences when APOE is present in circulation at higher-than-normal concentrations. Though physiological levels of wild-type APOE promote atherogenic lipoprotein clearance, overexpression has been shown to result in severe hypertriglyceridemia (25). This suggests that while APOE plays an essential role in lipid homeostasis under normal conditions, alterations in its expression or function may lead to lipid dysregulation, which could have significant implications for metabolic diseases such as hyperlipidemia and atherosclerosis.

Based on this knowledge, a bioengineered variant of APOE4, termed APOE4mut1, was developed (26). In this variant, the hydrophobic amino acids L261, W264, F265, L268, and V269 at the carboxy-terminal end were replaced with alanine. APOE4mut1 demonstrated significant capacity to clear atherogenic lipoproteins from circulation without triggering hypertriglyceridemia, even when present at higher than physiological levels (27). Physicochemical analysis revealed that the APOE4mut1 variant exhibits a more compact folding in three-dimensional space compared to wild-type APOE4 (27), which likely contributes to its enhanced biological properties. Indeed, functional data in animal models showed that, unlike wild-type APOE4, APOE4mut1 does not inhibit LpL activity in plasma and does not increase the rate of hepatic VLDL-triglyceride secretion (26). Furthermore, APOE4mut1 effectively clears plasma cholesterol and normalizes plasma triglyceride levels even at supraphysiological concentrations, while promoting the formation of high-density lipoprotein (HDL) particles (26, 28).

The primary objective of the present study was the investigation of the effects of APOE4mut1 on adipose tissue metabolism and its influence on the development of diet-induced obesity. To achieve this, we employed the 3T3-L1 preadipocyte cell line and three distinct sets of experiments using mouse models. Our results highlight a beneficial role of APOE4mut1 in the development of diet-induced obesity by reducing the extent of differentiation of 3T3-L1 preadipocytes into mature adipocytes and promoting a direct positive effect on white and adipose tissue mitochondrial metabolic activation in experimental mice. Our findings support that, in addition to their role in plasma triglyceride metabolism, amino acids L261, W264, F265, L268, and V269 in helix 8 of APOE4 critically regulate adipose tissue metabolism and lipid homeostasis.

## Materials and methods

### Cells

The AD293 cell line, a series of human embryonic kidney cells derived from the HEK293 cell line, was used for the development of adenoviruses (29). The 3T3-L1 fibroblasts were employed to study the mechanisms mediating adipocyte differentiation under the expression of apolipoproteins in vitro (30). AD293 and 3T3-L1 cell lines were purchased from Agilent Technologies and American Type Culture Collection (ATCC), respectively. All cell handling was conducted in a laminar flow cabinet sterilized with ultraviolet radiation and a 70% ethanol solution. Furthermore, the development of adenoviruses was carried out in a specially designed cell culture room that complies with the required safety regulations. Cell growth took place in an incubator with an atmosphere enriched with 5% CO_2_, maintained at a constant temperature of 37°C and under humidified conditions.

### Differentiation of 3T3-L1 fibroblasts into mature adipocytes

To induce the differentiation of fibroblasts into mature adipocytes, it is necessary to inhibit the proliferation of preadipocytes at the G1/S phase boundary following contact inhibition. Maximum cell-to-cell contact was achieved during culture when the number of 3T3-L1 preadipocytes exceeded 100% confluency, forming multilayered sheets. Subsequently, the cells were transduced with adenoviruses for 48 h, followed by the addition of an equal volume of differentiation medium (Dulbecco's Modified Eagle Medium, DMEM) high glucose supplemented with 10% fetal bovine serum (FBS), 1% Penicillin/Streptomycin (100×), 1 μM dexamethasone, 0.5 mM 3-Isobutyl-1-methylxanthine (IBMX), and 2 μg/ml insulin) (Day 0). The cells were incubated for 48 h, allowing them to complete two full cycles of cell division (mitotic clonal expansion). After the initial 48 h, the differentiation medium was replaced with maintenance and growth medium (DMEM high glucose, 10% FBS, 1% Penicillin/Streptomycin (100×), and 1 μg/ml insulin) for 3T3-L1 mature adipocytes. The maintenance medium was renewed every 48 h. Differentiation was completed between 8 days (early differentiation) and 16 days (mature differentiation) post-Day 0. Successful differentiation was confirmed via microscopic observation, identifying lipid droplets within the adipocytes (30).

### Oil Red O staining

The percentage of differentiated cells was determined by staining them with Oil Red O, a dye that binds to the lipid droplets of mature adipocytes. For this purpose, at the end of the differentiation process, the cells were fixed by replacing the culture medium with an equal volume of 10% formalin solution after two washes with 1× phosphate-buffered saline (PBS). The cells were incubated for 30–60 min at room temperature and then stored at 4°C until staining. Following staining, the cells were observed under a light microscope and photographed, with the lipid droplets in the adipocytes appearing as vivid red spots.

### Elution of Oil Red O dye from adipocytes

To enable comparison between different experimental groups, the Oil Red O dye was eluted from the cells, with the amount of eluted dye reflecting the percentage of differentiated cells. Specifically, the cells were incubated with 99% isopropanol for 10 min on a shaking platform at room temperature. The volume of isopropanol added was proportional to the size of the cell culture flask. Next, 200 μl of the eluted solution from each culture was transferred in triplicate to individual wells of a 96-well microplate for photometric analysis. Optical absorbance was measured at 490 nm. As a blank (control), 99% isopropanol was used. Additionally, a standard curve was generated using serial dilutions of the staining solution in 99% isopropanol to confirm the linearity of the measurements. The results were calculated after normalizing the absorbance readings to the blank control and were expressed as percentages relative to the absorbance of reference cultures that were not transduced with adenoviruses (31).

### Determination of intracellularly accumulated triglycerides in mature adipocytes

To determine the levels of accumulated triglycerides in mature adipocytes, differentiation cultures were performed in triplicate for each experimental group. On the 16th day of differentiation, the maintenance medium was removed from each well, and the cells were washed twice with 1× PBS. The cells were then incubated with a homogenization solution (10M KOH and 100% ethanol in a 1:1 ratio), collected and further incubated at 65°C overnight. Subsequently, the pH of the solution was adjusted to 7 by adding 37% HCl, and the final volume of the solution was recorded. Triglyceride levels were determined using the Triglycerides FS kit (Diagnostic Systems, GmbH), following the manufacturer’s instructions. Results were expressed as milligrams (mg) of triglycerides per milligram of protein ± SEM for each experimental group. To calculate the protein content, the mean value of total protein measurements from analogous cultures that underwent total protein isolation was used.

### Real-time PCR analysis of gene expression

Total RNA was extracted from 3T3-L1 cultures infected with AdGFP, AdAPOE4 or AdAPOE4mut1 using TRIzol reagent (Invitrogen) according to manufacturer’s instructions. Reverse transcription was performed using the PrimeScriptTM RT reagent kit from Takara Bio Inc. (catalog number RR037A; Otsu). Real-time PCR was performed in a MicroAmp® 96-well reaction plate from Applied Biosystems (catalog number 4346906; Foster City, CA) using the KAPA SYBR® FAST Universal qPCR kit from Kapa Biosystems (catalog number KK4601) in an Applied Biosystems StepOnePlus™ cycler. The primers used are shown below. Primers were synthesized by Eurofins Genomics. Data were normalized for rps18 expression.

• *rps18*_F 5′-GTA ACC CGT TGA ACC CCA TT-3′ (20 bases)
• *rps18*_R 5′-CCA TCC AAT CGG TAG TAG CG-3′ (22 bases)
• *fasn*_F 5′-AGG TGG TGA TAG CCG GTA TGT-3′ (21 bases)
• *fasn*_R 5′-TGG GTA ATC CAT AGA GCC CAG-3′ (21 bases)
• *dgat1*_F 5′-GTG CCA TCG TCT GCA AGA TTC-3′ (21 bases)
• *dgat1*_R 5′-GCA TCA CCA CAC ACC AAT TCA G-3′ (22 bases)
• *ppar-gamma*_F 5′-GGA AGA CCA CTC GCA TTC CTT-3′ (21 bases)
• *ppar-gamma*_R 5′-GTA ATC AGC AAC CAT TGG GTC A-3′ (22 bases)
• *adiponectin*_F 5′-GATCTGACGACACCAAAAGGGCTC-3′ (24 bases)
• *adiponectin*_R 5′-GGCTCTCCTTTCCTGCCAGGGG-3′ (22 bases)
• *fabp4*_F 5′-TGCCTTTGTGGGAACCTGGAAG-3′ (22 bases)
• *fabp4*_R 5′-CCACCACCAGCTTGTCACCATC-3′ (22 bases)
• *fabp5*_F 5′-TGGGAAGATGATCGTGGAGTGTG-3′ (23 bases)
• *fabp5*_R 5′-GGGATCCTGAAGAATACCAGAGAGC-3′ (25 bases)
• *lpl*_F 5′-ACCCCCTAGACAACGTCCACC-3′ (22 bases)
• *lpl*_R 5′-AGCTGGTCCACGTCTCCGAGT-3′ (22 bases)

### Expansion and purification of recombinant adenoviruses

Following plaque identification and isolation, adenoviruses ΑdGFP, AdAPOE4 and AdAPOE4mut1 were expanded in AD293 cells and then purified by double CsCl ultracentrifugation, followed by dialysis and titration of the recombinant adenoviruses, as described previously (32).

## Animals

Male C57BL/6 mice and male mice expressing human APOE4 (APOE4_knock-in_), approximately 24 weeks old, were allowed unrestricted access to water under a 12-h light/dark cycle (7:00 a.m.–6:59 p.m. light). C57BL/6 and APOE4_knock-in_ mice were purchased from the Jackson Laboratory and Taconic Farms, respectively. Groups of approximately four mice each were formed, and special care was taken to include in the same group animals with similar initial levels of plasma lipids and glucose, as well as body weight. No randomization was needed since all mice have an identical genetic background.

The C57BL/6 mice were distributed into six groups, three out of which were fed a high-fat diet (Μucedola SRL, Milano, Italy: 17.3% protein, 48.5% carbohydrates, 21.2% fat, 0.2% cholesterol, 4.5 kcal/g) for 8 weeks and the other three for 24 weeks. Then, mice were infected with either an adenovirus expressing the wild-type human APOE4 or an adenovirus expressing the biotechnologically modified form of human APOE4, APOE4mut1. Following infection, mice were maintained on high-fat diet. For each experiment, one group of mice was infected with a control adenovirus expressing only green fluorescent protein (GFP). In the same fashion, APOE4_knock-in_ mice were divided into two groups and fed the same high-fat diet for 4 weeks. Then mice were infected either with the adenovirus expressing APOE4mut1 or the control adenovirus and maintained under high-fat diet. All adenovirus doses were 2 × 10^9^ pfu, and the infection was performed by intravenous injection via the tail vein. Five days after the infection, mice were fasted for 16h, and following sacrifice, blood and tissues were collected for further analyses.

All animals were individually caged during the course of the study. No animal was excluded from the study. Sample size was determined based on the desired power of statistical analysis, using an online statistical tool (http://www.stat.ubc.ca/∼rollin/stats/ssize/n2.html) using mu1 = 0.75, mu2 = 1, σ = 0.1, a = 0.05, and desired power of analysis = 0.95. All animal experiments were conducted according to the EU guidelines of the Protocol for the Protection and Welfare of Animals. The work was authorized by the Laboratory Animal Centre committee of The University of Patras Medical School and the Veterinary Authority of the Prefecture of Western Greece (Authorization number ΠΔΕ/ΔΚ/44966/198 approved on 20 February 2023).

### Fractionation and purification of plasma lipoproteins

Pooled plasma of 0.4 ml from each mouse group was fractionated by KBr density gradient ultracentrifugation (KBr, 1.23 g/ml over 1.21 g/ml over 1.063 g/ml over 1.019 g/ml over saline), as described previously (33). Ten lipoprotein fractions were collected and purified by dialysis.

### Biochemical determination of plasma and lipoprotein lipid levels and plasma glucose levels

Plasma and lipoprotein total cholesterol and triglycerides levels were determined spectrophotometrically using the DiaSys Cholesterol FS kit (cat# 113009910021, Diagnostic Systems GmbH) and DiaSys Triglycerides FS kit (cat# 157109910021, Diagnostic Systems GmbH), respectively, according to the manufacturers’ instructions. Biochemical determination of plasma glucose levels was performed spectrophotometrically with a DiaSys Glucose FS kit (cat# 125009910021, Diagnostic Systems GmbH) according to the manufacturer's instructions.

### Isolation of intact mitochondria from adipose tissue

For the isolation of mitochondria from the white (WAT) and brown adipose tissue (BAT), the whole tissue was mechanically homogenized, and mitochondria were isolated by sequential centrifugations, as described previously (34). The protein concentration of each mitochondrial sample was determined using the DC™ Protein Assay Kit (cat# 500-0116; Bio-Rad).

### Western blot analysis

For the semiquantitative measurement of murine and human protein levels in the plasma and the lipoprotein fractions of the mice, SDS-PAGE (sodium dodecyl sulfate-polyacrylamide gel electrophoresis) was performed, followed by Western blot, using the following antibodies: Rabbit anti-Human and anti-Mouse APOE (D7I9N, cat# 10197, Cell Signaling), Goat anti APOB-48/100 HRP (cat# K34005G, Meridian Life Science), Rabbit anti-Mouse β-tubulin(cat# # 2146, Cell Signaling), Rabbit anti-Mouse Cytc (cat# 4272, Cell Signaling), Rabbit anti-Mouse Ucp1 (E9Z2V, cat#72298, Cell Signaling), Rabbit anti-Mouse Cox4 (cat# 4844, Cell Signaling), and Goat anti-rabbit IgG-HRP (cat#7074, Cell Signaling). SDS-PAGE of pure mitochondrial extracts was performed using 6 μg protein per sample for BAT and 15 μg protein per sample for WAT. Semiquantitative determination of the relative protein amounts was performed by ImageJ free software (Fiji, Version 1.52a, Wayne Rasband). Since there is no housekeeping protein that could be used as an internal standard, Western-blot analysis of apolipoproteins in culture medium or in the various lipoprotein fractions isolated from the plasma of mice was normalized based on sample volume (i.e. an equal amount of volume from each sample was loaded and analyzed by Western blot).

### Statistical analysis

All data sets were tested using the Kolmogorov-Smirnov and the Shapiro-Wilk tests and were treated with parametric (*P* > 0.1) or non-parametric tests (*P* < 0.1) according to their deviation from normality. Our analysis was performed using one-way ANOVA, when one dependent variable was tested, and two-way ANOVA, when more than one dependent variables were tested. The post hoc test used in our analysis was Tukey’s test. Data are reported as mean ± SEM, and a statistically significant *P*-value is set at <0.05 with a 95% confidence interval. ∗ = *P* value < 0.05, ∗∗ = *P* value < 0.005 and ∗∗∗ = *P* value < 0.0005. All statistical tests were performed using the GraphPad Prism 8.0.1 software.

## Results

### Effects of APOE4 or APOE4mut1 expression on 3T3-L1 adipocyte differentiation

To study the effects of APOE4 and APOE4mut1 on the differentiation of preadipocytes to mature adipocytes, the 3T3-L1 cell line was selected. For this purpose, cells were infected with a recombinant adenovirus expressing either *APOE4* (AdAPOE4) or *APOE4mut1* (AdAPOE4mut1), and their differentiation into mature adipocytes was induced for 16 days. In the control group, the infection was performed with an adenovirus expressing only the green fluorescent protein (AdGFP).

**Fig. 1:**
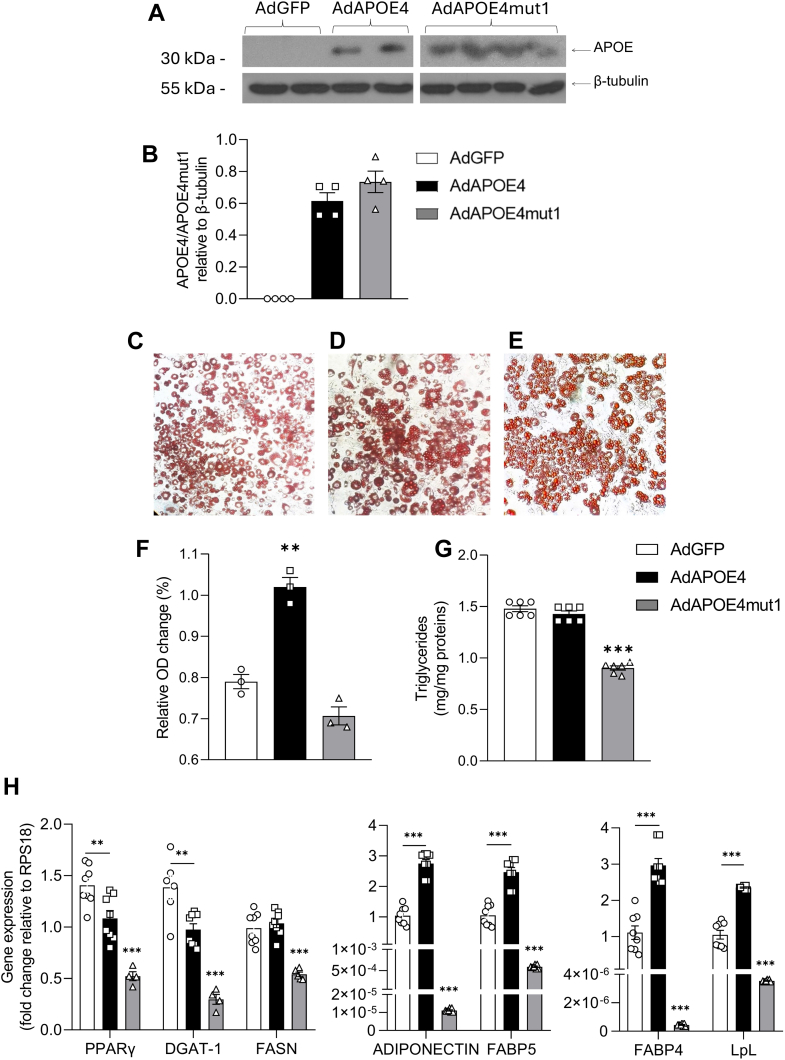
Effects of APOE4 and APOE4mut1 on 3T3-L1 adipocyte differentiation. A: Representative Western blot analysis for APOE4, APOE4mut1 in samples of culture medium collected on the 10th day of 3T3-L1 cell differentiation as well as β-tubulin in the respective cell lysates. Samples for APOE were analysed on two separate blots. B: Semiquantitative analysis of APOE4 and APOE4mut1 levels shown in the blots of panel A, normalized for β-tubulin levels (C–E) Representative images at 10× magnification of differentiated 3T3-L1 adipocytes after Oil Red O staining. The cells were induced to differentiate after infection with: (C) AdGFP, (D) AdAPOE4, (E) AdAPOE4mut1. F: Relative absorbance of Oil Red O dye, eluted from cultures infected with recombinant adenovirus. The values were derived from the average of at least three cultures for each adenovirus used. The data were analyzed using one-way ANOVA, and the values are expressed as Mean ± SEM. G: Effect of APOE4 and APOE4mut1 on triglyceride accumulation in mature 3T3-L1 adipocytes. The values for each culture were normalized to the total protein levels of the cells present in the culture. The analysis of the results was performed using one-way ANOVA, and the values are presented as Mean ± SEM. H: mRNA expression levels of the lipogenic genes *pparγ*, *dgat1*, and *fasn* as well as *pparγ* effector genes *adiponectin*, *fabp4*, *fabp5*, and *lpl* in differentiated 3T3-L1 adipocytes expressing APOE4 and APOE4mut1. The results were normalized to the expression levels of the housekeeping *rps18* gene. The analysis was performed using two-way ANOVA, and the values are presented as Mean ± SEM. ∗∗ = *P* < 0.005 and ∗∗∗ = *P* < 0.0005, n = 3 per group. Unless indicated by a horizontal line between two groups, statistical significance refers to differences among all test groups.

The successful infection of the cultured cells by the adenoviruses was confirmed by Western blot of the culture medium from AdGFP, AdPOE4, and AdAPOE4mut1 infected cells (Fig. 1A). Normalization of APOE expression to β-tubulin showed no differences between the two forms of APOE (Fig. 1A, B). The effect of APOE4 and APOE4mut1 expression during adipocyte differentiation was qualitatively assessed after Oil Red O staining on day 16 of differentiation. The cultures were then photographed after observation under a light microscope, confirming successful differentiation. Based on visual observation, the expression of APOE4 during differentiation appeared to positively influence adipocyte development compared to the modified APOE4mut1, which had an inhibitory effect with a reduction in the number of adipocytes observed in the infected cultures (Fig. 1B–D). The degree of differentiation in each culture was determined semi-quantitatively after elution of the Oil red O dye and photometric measurement as described in methods. Since a higher degree of cell differentiation in a culture results in larger lipid droplets present in mature adipocytes, the amount of bound dye present in each cell culture is a direct measure of the extent of adipocyte differentiation.

Therefore, after eluting Oil Red O in a given volume of extraction solution for each culture, the higher the optical absorbance of each dye solution, the higher the degree of differentiation. As shown in Fig. 1E, the results of this assay show that 3T3-L1 preadipocytes expressing APOE4 are differentiated to a greater extent than control cultures, as there was a significantly higher presence of dye in cells infected with AdAPOE4 compared to the cells infected with AdGFP. On the other hand, cells expressing APOE4mut1 show a degree of differentiation similar to that of control AdGFP-infected cultures and much lower than that of AdAPOE4-infected cultures.

Lipid droplets are the main organelle for lipid storage within adipocytes, with triglycerides being the primary form of lipid storage. During lipogenesis, the lipid droplets in adipocytes increase in size, either by fusing with each other or by gradually accumulating more lipids, often occupying nearly the entire cytoplasmic area. Therefore, effective differentiation of 3T3-L1 cells should be evaluated by considering both the number of mature adipocytes and their lipid content. To this end, the levels of intracellularly accumulated triglycerides in mature adipocytes on the 16th day of differentiation were determined. As shown in Fig. 1F, the expression of APOE4 during differentiation did not cause a significant increase in triglyceride accumulation in adipocytes compared to the control (AdGFP) culture, although staining with Oil-Red O indicated an increase in differentiation due to APOE4 expression. In contrast, the expression of the modified APOE4mut1 resulted in a significant reduction in triglyceride accumulation within the adipocytes (*P* ≤ 0.0001), compared to both AdGFP and AdAPOE4 infected cultures.

To further investigate the effects of APOE4 and APOE4mut1 on lipogenesis in 3T3-L1 cells, we analyzed the mRNA expression levels of the key lipogenic genes *pparγ* (Peroxisome Proliferator-Activated Receptor gamma), *dgat1* (DiacylGlycerol O-AcylTransferase 1) and *fasn* (Fatty Acid Synthase) using qPCR. The presence of either APOE4 or APOE4mut1 during 3T3-L1 preadipocyte differentiation led to a reduction in *pparγ* mRNA expression compared to control AdGFP-infected cultures (Fig. 1G). However, APOE4 caused a moderate ∼30% decrease, while APOE4mut1 led to a much more potent ∼60% reduction (Fig. 1G). A similar trend was observed for *dgat1* mRNA levels, where APOE4 caused a ∼35% decrease, whereas APOE4mut1 resulted in a more severe ∼85% reduction (Fig. 1G). In contrast, *fasn* mRNA expression was unaffected by APOE4 compared to the control group (Fig. 1G). However, APOE4mut1 significantly reduced *fasn* mRNA levels by approximately 50% (Fig. 1G).

Consistent with the reductions observed in *pparγ* expression, we next examined several established *pparγ* effector genes, including *adiponectin*, *fabp4* (Fatty Acid Binding Protein 4), *fabp5* (Fatty Acid Binding Protein 5), and *lpl*. APOE4 moderately increased adiponectin mRNA levels relative to control cultures, whereas APOE4mut1 caused a potent suppression, reducing *adiponectin* mRNA expression to very low levels. A similar pattern was observed for *fabp4, fabp5*, and *lpl* mRNA expression. These data support that APOE4mut1 not only reduces *pparγ* levels but also strongly impairs its downstream transcriptional program, leading to a broad suppression of additional key adipogenic genes.

APOE4mut1 expression in the plasma of C57BL/6 mice fed high-fat diet for 8 weeks has a positive effect on lipid and glucose levels.

In the next set of experiments, we aimed to examine the in vivo effects of APOE4 and APOE4mut1 on adipose tissue metabolic activation in C57BL/6 mice. After eight weeks on a high-fat diet, mice were divided into three groups. The first two groups received 2 × 10^9^ pfu/mouse of either AdAPOE4 or AdAPOE4mut1, while the third group received 2 × 10^9^ pfu/mouse of AdGFP to account for any non-specific effects (i.e. inflammatory, etc) of the viral infection. No difference in food or water consumption was noted among groups during the course of the study (not shown).

**Fig. 2:**
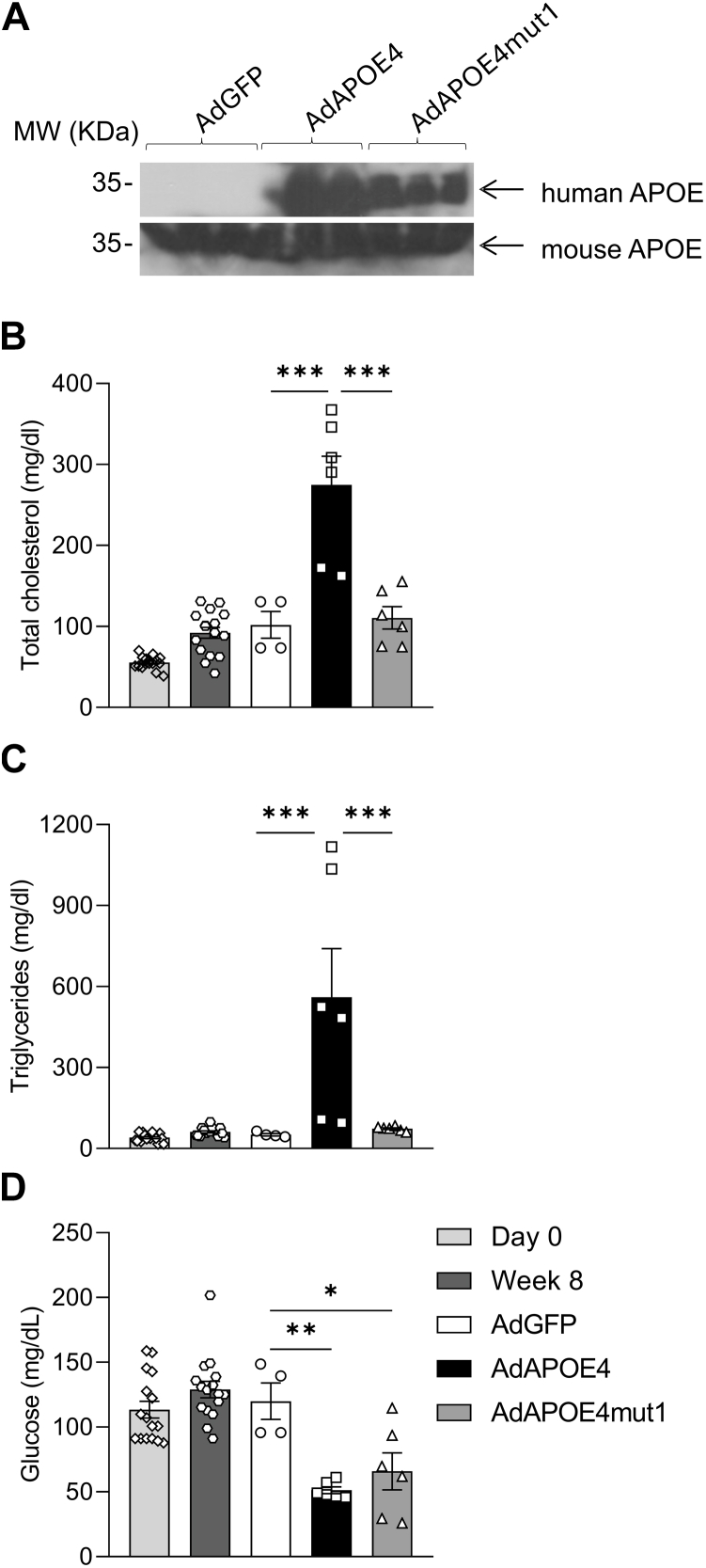
Plasma lipid and glucose levels of C57BL/6 mice fed high-fat diet for 8 weeks and then infected with the adenoviruses AdGFP, AdAPOE4, and AdAPOE4mut1. A: Representative Western blot of human and murine APOE five days after infection of mice with the adenoviruses, (B) plasma total cholesterol, (C) plasma total triglyceride, and (D) plasma glucose levels five days after infection of mice with the adenoviruses. The results were analyzed using one-way ANOVA. Values are expressed as Mean ± SEM. ∗ = *P* < 0.05, ∗∗ = *P* < 0.005 and ∗∗∗ = *P* < 0.0005, n = 3 per group.

Western blot analysis of plasma samples collected 5 days post-infection confirmed the expression of human APOE in mice infected with AdAPOE4 and AdAPOE4mut1 (Fig. 2A). As expected, no detectable levels of human APOE were observed in the plasma of mice infected with the control AdGFP adenovirus (Fig. 2A). In addition, analysis of the endogenous murine APOE did not reveal significant differences among groups (Fig. 2A).

Fasting plasma lipid analysis shows that the expression of the human APOE4 leads to significantly increased levels of both total cholesterol (Fig. 2B) and triglycerides (Fig. 2C). In contrast, the expression of APOE4mut1 maintains both plasma cholesterol and triglyceride levels within physiological and comparable to those of the control group (AdGFP) (Fig. 2B, C).

The significantly reduced plasma lipids observed in mice expressing APOE4mut1 compared to mice expressing APOE4 led us to measure glucose levels in the fasting plasma samples of the three groups of experimental animals. Our results show that feeding mice a high-fat diet for 8 weeks did not significantly affect fasting glucose levels. Following infection of mice with the different adenoviruses, both APOE4 and APOE4mut1 led to improved fasting glucose levels. In AdGFP-infected mice, glucose levels remained unchanged and comparable to those of week 0 and week 8 prior to infection (Fig. 2D).

**Fig. 3:**
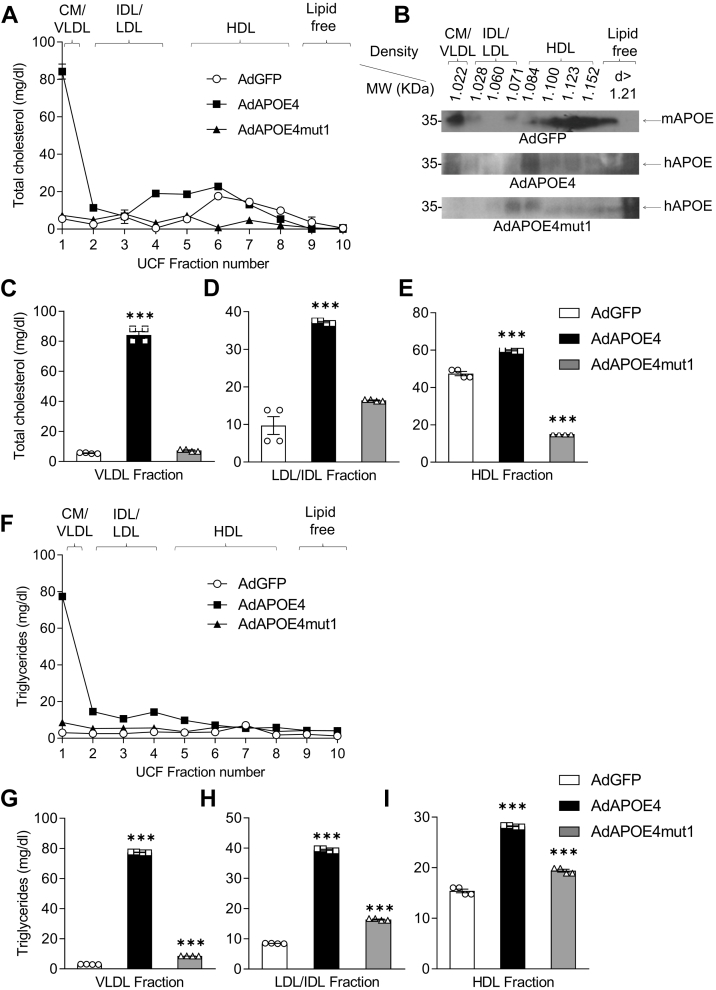
Plasma lipoprotein lipid levels of C57BL/6 mice fed high-fat diet for 8 weeks and then infected with the adenoviruses AdGFP, AdAPOE4, and AdAPOE4mut1. Lipoproteins were fractionated by KBr density gradient ultracentrifugation. A, C–E: Total cholesterol and (F–I) triglyceride levels of lipoprotein fractions 5 days after infection of mice with the adenoviruses. Panel B shows the murine APOE, human APOE4 and APOE4mut1 distribution among lipoprotein fractions. The results were analyzed using one-way ANOVA, and values are expressed as Mean ± SEM. ∗∗∗ = *P* < 0.0005, n = 3 per group. Statistical significance refers to differences among all test groups.

Fractionation of plasma lipoprotein and analysis of cholesterol and triglyceride levels in the lipoprotein fractions revealed that the significant increase in cholesterol levels observed in the plasma of experimental animals expressing APOE4 is associated with an increase in the cholesterol levels of all lipoprotein classes (Fig. 3A, C–E). Furthermore, it appears that the expression of APOE4mut1 leads to a significant reduction in the cholesterol levels of all lipoprotein fractions (Fig. 3A, C–E).

Analysis of triglyceride levels in lipoprotein fractions shows that the significant increase observed in the plasma of experimental animals after APOE4 expression is associated with an increase in triglyceride levels mainly in VLDL and to a much lesser extent in IDL (intermediate density lipoprotein) and LDL (low density lipoprotein) (Fig. 3F–I). Again, expression of APOE4mut1 maintains triglyceride levels within physiological levels in all lipoprotein classes and comparable to those of mice infected with the control adenovirus AdGFP (Fig. 3F–I).

As shown in Fig. 3B, APOE4mut1 is restricted to the HDL and LDL fractions, whereas murine APOE and human APOE4 are also present in the VLDL fraction. This distribution pattern may underline the divergent lipid profiles observed in APOE4-and APOE4mut1-expressing mice. Because APOE inhibits LpL, the presence of APOE4 on VLDL in AdAPOE4-infected mice may impair VLDL-TG lipolysis and slow remnant clearance, whereas in AdAPOE4mut1-infected mice—where APOE4mut1 is absent from VLDL—these processes are likely less constrained.

### Effect of APOE4 and APOE4mut1 expression on metabolic activation of adipose tissue in experimental animals fed high-fat diet for 8 weeks

To investigate the effect of APOE4 and APOE4mut1 on the metabolic activation of adipose tissue, mice were fed a western-type diet for 8 weeks and then infected for an additional five days with AdGFP, AdAPOE4 and AdAPOE4mut1. Then, purified mitochondria were isolated from WAT and BAT and western-blot analysis of mitochondrial extracts was performed for Ucp1 and CytC in relation to COx4 as surrogate biochemical markers of non-shivering thermogenesis and oxidative phosphorylation, respectively.

**Fig. 4:**
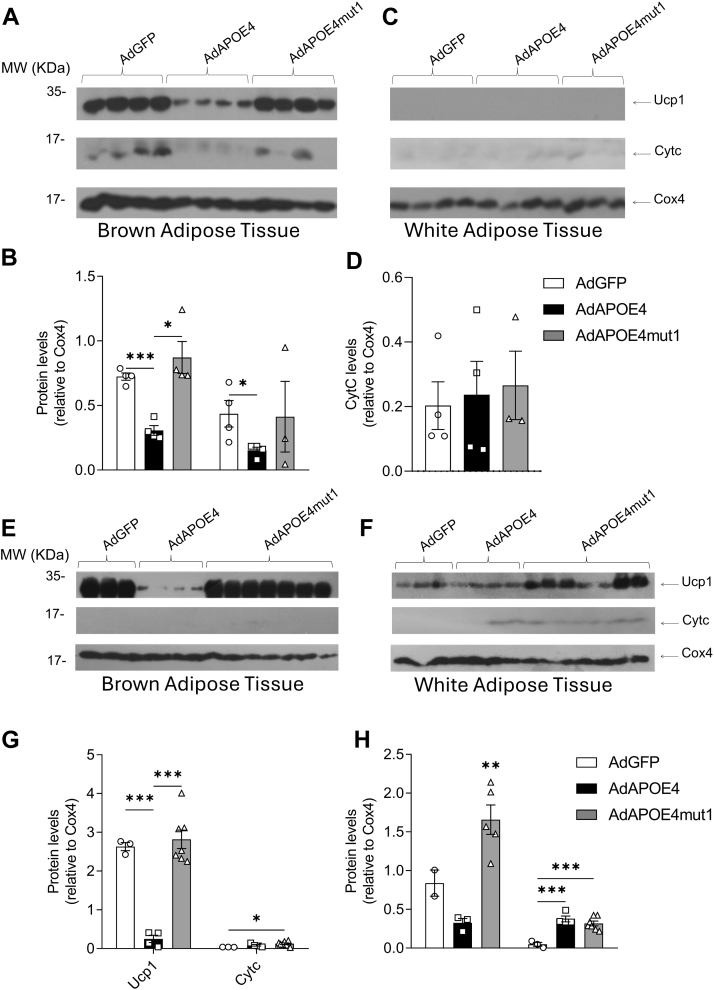
Western Blot analysis for the determination of Cytc and Ucp1 levels in relation to Cox4 in mitochondrial fractions from BAT (A, E) and WAT (C, F) isolated from the experimental animals, fed high-fat diet for 8 weeks (A, C) or 24 weeks (E, F), 5 days after infection with the adenoviruses AdGFP, AdAPOE4, and AdAPOE4mut1. Semi-quantitative determination for BAT (B, G) and WAT (D, H) mitochondrial protein levels based on the corresponding western blots was performed using Image J free software. For the 8-weeks treatment, the results were analysed using two-way ANOVA, and values are expressed as Mean ± SEM (n = 4 per group). For the 24-weeek treatment, data are presented as Mean ± SEM and were analyzed using two-way ANOVA (n = 3 for AdGFP, n = 4 for AdAPOE4, and n = 7 for AdAPOE4mut1 group). Unless indicated by a horizontal line between two groups, statistical significance refers to differences among all test groups. ∗ = *P* < 0.05, ∗∗ = *P* < 0.005 and ∗∗∗ = *P* < 0.0005.

Expression of APOE4 led to a marked reduction in mitochondrial non-shivering thermogenesis in BAT compared to the control AdGFP-infected group, as indicated by Ucp1 levels (Fig. 4A, B). In contrast, the presence of APOE4mut1 does not appear to interfere with this process (Fig. 4A, B). Additionally, the presence of APOE4 resulted in a significant reduction in oxidative phosphorylation in the mitochondria of BAT, as shown by CytC levels, while APOE4mut1 maintained CytC at levels similar to those of the AdGFP group (Fig. 4A, B).

Similar analysis in WAT did not reveal differences in oxidative phosphorylation among the three experimental groups, as indicated by CytC levels (Fig. 4C, D). Ucp1 levels were not detectable in WAT (Fig. 4C).

### Effect of APOE4 and APOE4mut1 expression on metabolic activation of adipose tissue in experimental animals fed high-fat diet for 24 weeks

In the next set of experiments, mice were fed a western-type diet for 24 weeks and then infected for an additional 5 days with AdGFP or AdAPOE4 or AdAPOE4mut1 adenoviruses. Purified mitochondria were again isolated from WAT and BAT of mice, and Western-blot analysis of mitochondrial extracts was performed for Ucp1 and CytC in relation to COx4. No difference in food or water consumption was noted among groups during the course of the study (not shown).

Our data show that expression of APOE4 led to a reduction in non-shivering thermogenesis, indicated by the reduced levels of Ucp1 in BAT, compared to the control group (Fig. 4E, G). A similar effect was observed in WAT, but it did not reach statistical significance (Fig. 4F, H). In contrast, when APOE4mut1 was expressed, no suppression of non-shivering thermogenesis compared to the control group was noted in BAT, whereas a notable increase in Ucp1 levels (Fig. 4G, H) was found in WAT, indicating the conversion of WAT into BRITE. Both APOE4 and APOE4mut1 increased mitochondrial CytC levels in WAT (Fig. 4H)

The effect of APOE4mut1 is dominant in experimental animals expressing endogenous wild-type APOE4.

**Fig. 5:**
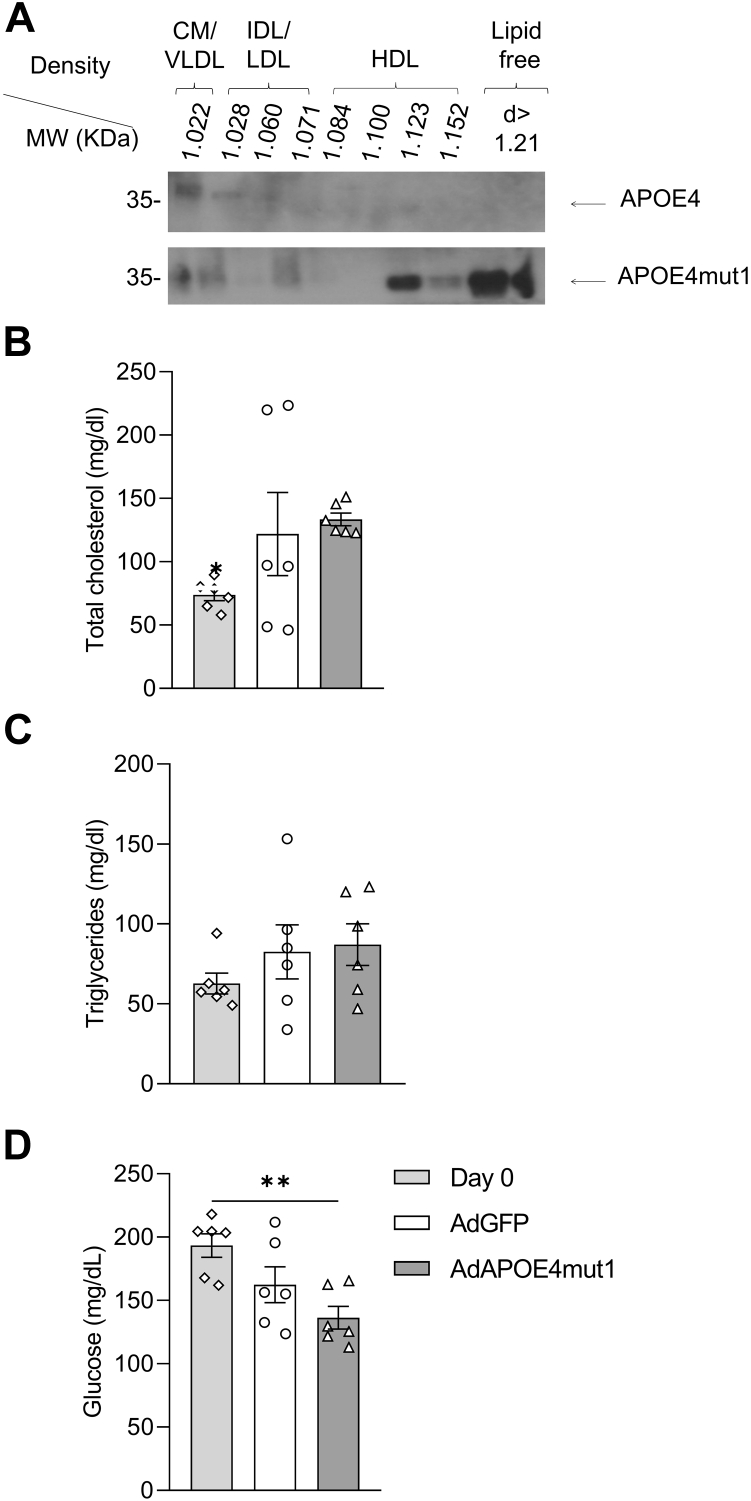
Wild-type APOE4 and APOE4mut1 distribution among lipoprotein fractions (A) and changes in cholesterol (B), triglyceride (C), and glucose (D) levels in the plasma of APOE4_knock-in_ mice fed high-fat diet for 4 weeks, under the influence of adenovirus expression of AdGFP (expressing endogenous APOE4) and AdAPOE4mut1 (expressing APOE4mut1). The analysis of the results was performed with one-way ANOVA. Values are presented as Mean ± SEM. ∗ = *P* < 0.05, n = 3 per group. Unless indicated by a horizontal line between two groups, statistical significance refers to differences among all test groups.

To assess the potential dominance of the positive effects of APOE4mut1 over the negative effects of wt APOE4 on the metabolic activation of BAT and WAT, in the next set of experiments, we examined the impact of APOE4mut1 on mitochondrial Ucp1 and Cytc levels in the presence of endogenously expressed wild-type APOE4. For this purpose, we used APOE4_knock-in_ mice that express human APOE4 instead of murine APOE. Two groups were formed and fed a high-fat diet for 4 weeks. After 4 weeks, one group was administered 2 × 10^9^ pfu/mouse AdGFP, while the other received 2 × 10^9^ pfu/mouse AdAPOE4mut1. Five days after infection, the mice were euthanized, and blood samples and tissues were collected for analysis. Figure 5A shows APOE4 and APOE4mut1 lipoprotein distribution in the respective groups.

Mice infected with AdGFP showed a modest increase in plasma cholesterol levels, though no significant change was noted in plasma triglyceride levels five days post-infection. Similar plasma lipid levels were noted for mice infected with AdAPOE4mut1 (Fig. 5B, C). A statistically significant reduction in plasma glucose levels was also noted in mice expressing APOE4mut1 (Fig. 5D), consistent with the findings in C57BL/6 mice (Fig. 2D).

**Fig. 6:**
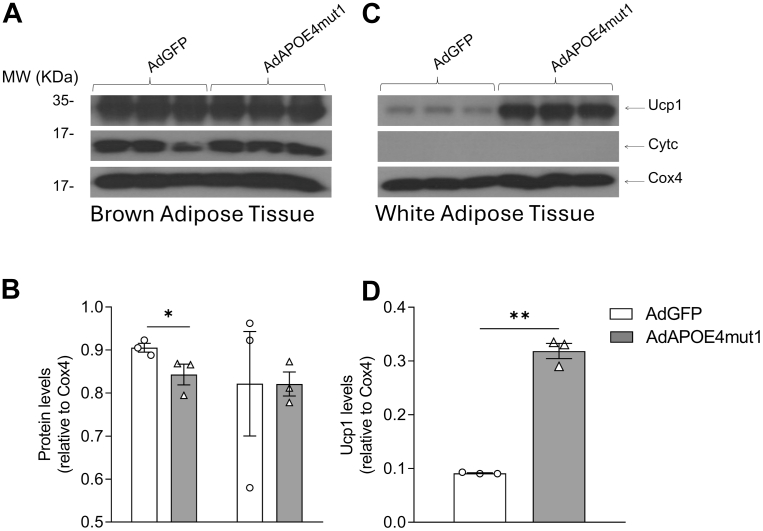
Western Blot analysis for the determination of Cytc and Ucp1 levels in relation to Cox4 in mitochondrial fractions of BAT (A, B) and WAT (C, D) isolated from APOE4_knock-in_ mice, 5 days after infection with AdGFP and AdAPOE4mut1 adenoviruses, and 4 weeks after high-fat diet administration. Semi-quantitative determination was performed using Image J free software. Results were analysed using two-way ANOVA, and values are expressed as Mean ± SEM. ∗ = *P* < 0.05, ∗∗ = *P* < 0.005, n = 3 per group.

Interestingly, expression of APOE4mut1 led to a striking increase in Ucp1 levels in purified WAT mitochondria, a surrogate biochemical marker of non-shivering thermogenesis (35), suggesting increased thermogenesis and transdifferentiation into BRITE (Fig. 6), a process associated with weight loss. CytC levels were very low and unaffected by APOE4mut1 expression. This result supports that the stimulatory effect of APOE4mut1 on non-shivering thermogenesis is dominant over the inhibitory effect of APOE4 that we observed in this process in our previous experiments (Fig. 4). When similar analysis was performed in isolated BAT mitochondria, Ucp1 and CytC levels were found unaffected, suggesting that expression of APOE4mut1 has no significant impact on non-shivering thermogenesis or oxidative phosphorylation in this tissue (Fig. 6).

## Discussion

Our study highlights the pivotal role of amino acid residues L261, W264, F265, L268, and V269 in helix 8 of human APOE in adipose tissue metabolic activity, providing new insights into potential therapeutic strategies for morbid obesity using APOE4mut1, where these five hydrophobic amino acid residues have been replaced by Alanine.

In vivo administration of recombinant adenovirus results to hepatic expression of APOE because of the natural tropism of the adenovirus. Therefore, to compare the effects of adipocytic APOE4 and APOE4mut1 expression on preadipocyte to adipocyte differentiation, we turned to in vitro studies using 3T3-L1 cells. It should be noted that, in contrast to the studies in 3T3-L1 cultures, where APOE4 and APOE4mut1 were expressed by the infected cells, the in vivo results of AdAPOE4 and AdPOE4mut1 infection on BAT and WAT are due to circulating hepatically expressed APOE.

Our in vitro experiments demonstrated that APOE4mut1 suppresses adipocyte differentiation and triglyceride accumulation in 3T3-L1 preadipocytes, unlike wild-type APOE4. To assess lipid levels in the tested cultures, we used two approaches: a) Oil Red O staining of differentiated cells followed by dye extraction and quantification, and b) direct measurement of triglyceride content in differentiated cells. APOE4 expression led to increased Oil Red O staining despite similar cellular triglyceride levels in AdGFP-infected cells, suggesting the presence of more and smaller lipid droplets. Similarly, APOE4mut1 expression resulted in comparable Oil Red O staining despite significantly lower triglyceride levels compared to control cultures, again indicating a higher number of smaller lipid droplets. However, both methods suggest that, unlike APOE4, APOE4mut1 does not promote differentiation.

This was corroborated by reduced expression of lipogenic genes such as *pparγ*, *fasn*, and *dgat1*, as well *pparγ* effector genes such as *fabp4*, *fabp5*, *adiponectin* and *lpl*, suggesting that APOE4mut1 may exert anti-adipogenic effects at the cellular level. These findings align with the observed phenotype of smaller lipid droplets and reduced triglyceride accumulation, further supporting the notion that amino acid residues L261, W264, F265, L268, and V269 in the carboxy-terminal region of APOE4 play a critical role in lipogenesis.

In vivo, the expression of APOE4mut1 stimulated non-shivering thermogenesis in adipose tissues, a stark contrast to the inhibitory effects of wild-type APOE4. Notably, APOE4mut1 increased mitochondrial Ucp1 levels in white adipose tissue (WAT), following prolonged high-fat diet feeding conditions (24 weeks long), suggesting a role in promoting a conversion into a BAT-like phenotype in WAT (conversion into BRITE tissue). This shift in mitochondrial function underscores the ability of APOE4mut1 to enhance energy expenditure, thereby contributing to resistance against diet-induced obesity. The lack of effect on mitochondrial CytC levels suggests that the thermogenic effects are mediated specifically through uncoupled oxidative phosphorylation rather than overall mitochondrial oxidative phosphorylation. The absence of Ucp1 stimulation in mice fed a Western-type diet for only 8 weeks may suggest a potential threshold of stress following chronic exposure to dietary fat, beyond which WAT responsiveness to APOE4mut1 may be triggered. As a matter of fact, conversion of white into brown adipose tissue (BRITE) in mice is also triggered by feeding a high-fat diet (36), and our data raise the possibility that APOE4mut1 may potentiate this phenomenon. This possibility warrants further exploration.

Interestingly, in BAT, APOE4mut1 also increased Ucp1 expression, further supporting its role in thermogenesis. Notably, this stimulatory effect was obvious even after 8 weeks of feeding high fat diet. Contrasting this observation, the suppression of BAT thermogenic activity by wild-type APOE4 highlights the divergent roles of the two proteins in adipose tissue metabolism.

Additionally, the dominance of APOE4mut1 over wild-type APOE4 in APOE4_knock-in_ mice further strengthens its therapeutic potential, as it suggests that APOE4mut1 can override the detrimental effects of wild-type APOE4 on adipose tissue metabolism. Remarkably, just four weeks of high-fat diet feeding were sufficient to induce non-shivering thermogenesis in WAT in APOE4mut1-expressing mice. This finding contrasts with observations in C57BL/6 mice, where 24 weeks of high-fat diet feeding were required to elicit increased WAT Ucp1 expression in response to APOE4mut1. One possible explanation is that in the APOE4_knock-in_ mouse model, APOE4 induces a stress threshold more rapidly than murine APOE, beyond which WAT responsiveness to APOE4mut1 may be activated. Consequently, shorter periods of high-fat diet feeding of APOE4_knock-in_ mice more effectively induce a response to APOE4mut1 in WAT. The possibility that APOE4 triggers a diet-induced stress threshold much faster than murine APOE indicates another point of functional difference between the two proteins and deems further investigation.

Analysis of plasma lipid and lipoprotein levels in C57BL/6 mice infected with AdAPOE4 or AdAPOE4mut1 showed that eexpression of APOE4 was associated with increased plasma cholesterol and triglyceride levels, as expected (26, 37). In contrast, expression of APOE4mut1 in these mice maintained blood lipid levels within physiological, compared to those of the AdGFP control group. When the lipids in various lipoprotein fractions were analysed, it was found that the significant increase in plasma cholesterol and triglyceride levels in the AdAPOE4-infected group was associated with elevated cholesterol and triglyceride levels in all lipoproteins. In contrast, expression of APOE4mut1 resulted in reduced cholesterol levels in LDL and HDL and normal triglyceride levels in all lipoproteins. Another important observation was that both APOE4 and APOE4mut1 expression in C57BL/6 mice led to a reduction in blood glucose levels in C57BL/6 mice treated with the corresponding adenoviruses, compared to the group treated with AdGFP. Steady-state plasma levels of APOE4, APOE4mut1, as well as endogenous murine APOE were similar between groups, indicating that the observed differences between them were not a result of differences in total plasma APOE levels but rather the result of the C-terminal mutations present in APOE4mut1.

A similar reduction in plasma glucose levels by APOE4mut1 expression was also observed in APOE4_knock-in_ mice, while plasma cholesterol and triglyceride levels remained unaffected and within physiological levels.

## Conclusion and Future Directions

Our findings demonstrate that the carboxy-terminal region of APOE, specifically amino acids L261, W264, F265, L268, and V269, is critical for modulating the protein’s effects on lipid metabolism and adipose tissue activity. The engineered APOE4mut1 variant exhibits a unique ability to enhance adipose tissue thermogenesis, suppress lipogenesis, while significantly reduces plasma glucose and lipid levels. In contrast, to wild-type APOE4, the engineered variant APOE4mut1 retains its ability to clear cholesterol-rich lipoproteins while mitigating the hypertriglyceridemic effects of wild-type APOE4. The lack of inhibitory activity on LpL and stimulatory activity on hepatic VLDL secretion (26), confirmed once again in the present study, are key to this variant’s favorable lipid profile, positioning it as a promising therapeutic tool for metabolic syndrome. It is quite intriguing that hepatically expressed APOE or APOE4mut1 appear to influence biochemical surrogate markers of mitochondrial metabolic activity in adipose tissue. One possibility is that they alter the flux of lipids to this tissue, influencing substrate availability. Another possibility is that these apolipoproteins and their respective lipoproteins affect the biophysical properties of cellular membranes like fluidity, which may modify the responsiveness of this tissue to external stimuli through cell-surface receptors (38). Finally, they may mediate the delivery, to adipose tissue, of other bioactive lipids that can modify the metabolic state of this tissue.

One limitation of our study is that our data on mitochondrial metabolic activity are based on the evaluation of the biochemical markers Ucp1 and CytC in isolated mitochondria rather than functional data from respirometry. Another limitation of the study is the short duration of APOE4mut1 expression following adenovirus-mediated gene transfer. Future studies should focus on the long-term effects of APOE4mut1 expression in diverse metabolic contexts and its potential interactions with other pathways involved in energy balance and lipid and glucose metabolism. Together, these findings pave the way for novel strategies to a holistic targeting of metabolic disorders using precision-engineered variants of APOE.

## Data Availability

Data will be made available upon reasonable request.

## Conflict of Interest

The authors declare the following financial interests/personal relationships which may be considered as potential competing interests:

KYRIAKOS E KYPREOS reports financial support was provided by Andreas Mentzelopoulos Foundation. ANCA GAFENCU reposts financial support was provided by European Union (NextGenerationEU), through Romania’s National Recovery and Resilience Plan, PNRR/2022/C9/MCID/I8, contract no. 760060/23.05.2023 (#258-STROMA) VICTORIA MPARNIA reports a relationship with Hellenic ministry of Agriculture that includes: funding grants. GEORGIA KAKAFONI reports a relationship with Andreas Mentzelopoulos Foundation that includes: funding grants. MADALINA DIMITRESCU reports a relationship with Romanian Academy that includes: funding grants. MADALINA FENYO reports a relationship with Romanian Academy that includes: funding grants. If there are other authors, they declare that they have no known competing financial interests or personal relationships that could have appeared to influence the work reported in this paper.
